# Shift from flooding to drying enhances the respiration of soil aggregates by changing microbial community composition and keystone taxa

**DOI:** 10.3389/fmicb.2023.1167353

**Published:** 2023-05-12

**Authors:** Kai Zhu, Weitao Jia, Yu Mei, Shengjun Wu, Ping Huang

**Affiliations:** Key Laboratory of Reservoir Aquatic Environment, Chongqing Institute of Green and Intelligent Technology, Chinese Academy of Sciences, Chongqing, China

**Keywords:** soil respiration, soil aggregates, water regime changes, microbial community, co-occurrence network, keystone taxa

## Abstract

Changes in the water regime are among the crucial factors controlling soil carbon dynamics. However, at the aggregate scale, the microbial mechanisms that regulate soil respiration under flooding and drying conditions are obscure. In this research, we investigated how the shift from flooding to drying changes the microbial respiration of soil aggregates by affecting microbial community composition and their co-occurrence patterns. Soils collected from a riparian zone of the Three Gorges Reservoir, China, were subjected to a wet-and-dry incubation experiment. Our data illustrated that the shift from flooding to drying substantially enhanced soil respiration for all sizes of aggregate fractions. Moreover, soil respiration declined with aggregate size in both flooding and drying treatments. The keystone taxa in bacterial networks were found to be *Acidobacteriales*, *Gemmatimonadales*, *Anaerolineales*, and *Cytophagales* during the flooding treatment, and *Rhizobiales*, *Gemmatimonadales*, *Sphingomonadales*, and *Solirubrobacterales* during the drying treatment. For fungal networks, *Hypocreales* and *Agaricalesin* were the keystone taxa in the flooding and drying treatments, respectively. Furthermore, the shift from flooding to drying enhanced the microbial respiration of soil aggregates by changing keystone taxa. Notably, fungal community composition and network properties dominated the changes in the microbial respiration of soil aggregates during the shift from flooding to drying. Thus, our study highlighted that the shift from flooding to drying changes keystone taxa, hence increasing aggregate-scale soil respiration.

## 1. Introduction

Soil aggregates, the fundamental building blocks of soil structure, perform a vital function in SOC turnover and nutrient cycling by providing different habitats for microbial activity (Six et al., 2004; Wang et al., 2021). According to the hierarchical model, aggregates are classified as macroaggregates (> 0.25 mm) and microaggregates (< 0.25 mm; Tisdall and Oades, 1982). Different aggregate size classes have distinct roles in soil nutrient supply and retention by influencing soil biological processes and pore characteristics (Mangalassery et al., 2013; Chen et al., 2023), thereby leading to small-scale heterogeneity in SOC mineralization (i.e., soil respiration) (Wang et al., 2019). Macroaggregates generally comprise labile young SOC, predominantly originating from fresh SOC inputs, fungal hyphae, and plant residues (Six et al., 2004). However, the most recalcitrant SOC formed by microbial-induced bonding of clay particles and organometallic complexes is stored in microaggregates (Zhang et al., 2018). Although macroaggregates are considered to have higher soil respiration than microaggregates (Noellemeyer et al., 2008; Fernández et al., 2010; Bandyopadhyay and Lal, 2014), macroaggregates have been shown to reduce soil respiration in contrast to microaggregates in various studies (Drury et al., 2004; Sey et al., 2008) or the same among aggregate size fractions (Razafimbelo et al., 2008; Rabbi et al., 2014). These conflicting results suggest that further research is required on the regulatory mechanisms of aggregate-scale soil respiration.

Soil respiration is profoundly affected by soil water regimes (Luo and Zhou, 2006; Zhu et al., 2020, 2022a). The changes in water regimes, such as intensive rain after a long drought or drying after intensive flooding, are dominant factor regulating biogeochemical processes in soils (Bodner et al., 2013; Evans and Wallenstein, 2014). These changes have a vital impact on soil aggregate stability, nutrient cycling, and microbial community composition and activity (Denef et al., 2001; Jansson and Hofmockel, 2020). Various microbial communities may colonize a given environment because varied sizes of aggregates provide unique niches for them to thrive in (including aerobic and anaerobic environments, for example) (Trivedi et al., 2017). Microaggregates have a high fungal abundance and a low bacterial abundance (Gupta and Germida, 1988; Jiang et al., 2018; Wang et al., 2021). Microaggregates amass more recalcitrant carbon, which is more favorable to oligotrophs, while macroaggregates contain comparatively much labile carbon, which favors copiotrophs (Trivedi et al., 2017). Increased soil water content generally promotes bacterial abundance, whereas drought decreases bacterial activity (Johan et al., 1986; Meisner et al., 2013). Owing to their thick cell walls, fungi are insufficiently sensitive to changes in soil moisture (Holland and Coleman, 1987; Umair et al., 2020; Dacal et al., 2022). Therefore, soil bacteria and fungi may respond differently to water regime changes at the aggregate scale (Navas et al., 2021), thereby affecting soil respiration. However, at the aggregate scale, our knowledge of how soil microbe communities respond to changes in water availability is still rather restricted.

Riparian zones, or the transition zones between aquatic and terrestrial ecosystems, facilitate critical ecological functions, such as supplying corridors for species migration and improving biodiversity (Jones et al., 2010; de Sosa et al., 2018). Water-level fluctuations have a significant impact on riparian ecosystems’ functions (Leira and Cantonati, 2008). These fluctuations are the major drivers of water regime changes (the shift from aerobic to anaerobic environments), which substantially affects soil respiration, soil aggregates and soil microbial community structure (Fierer et al., 2003; Zhu et al., 2022a). Nevertheless, it is not clear how microbial community changes at the aggregate scale due to water regime changes and how these changes affect respiration.

To date, research on the impacts of water regime changes on soil respiration and its mechanisms have primarily focused on wet–dry cycles (Jarvis et al., 2007), rewetting (i.e., rainfall) after long-term drought (De Nijs et al., 2019), peatland drainage, and water level decline (Silvola et al., 1996; Danevčič et al., 2010). Among these studies, the focus has been on bulk soil. However, little is known about the impact of soil microbes on aggregate-scale soil respiration under water regime changes in the riparian zone. Filling this knowledge gap will improve our understanding of the SOC dynamics in water regime changes, which thus contributing to the realization of riparian ecosystem carbon sequestration and emission reduction targets.

The riparian zones of the Three Gorges Reservoir (TGR) are an excellent location for investigating the influence of soil microbes in soil respiration in the face of extreme shifts in the water regime. The water level of the TGR in China varies from 145 m to 175 m during summer and winter, respectively, due to dam activities, which results in the formation of a new riparian zone that is 349 km^2^ (Zhu et al., 2020). In the TGR riparian zone, yearly disruption from fluctuating water levels, soil erosion and deposition caused by periodical draining and flooding, and the presence of microorganisms all have the potential to significantly alter soil aggregation (Xiang et al., 2018; Zhu et al., 2022a). She et al. (2022) recently reported that water regime changes result in distinct microbial community compositions and functions between the drainage and flooding periods, thereby controlling CH_4_ and CO_2_ emissions in the TGR. Zhu et al. (2022a) reported that in the riparian zone of the TGR, intense wet–dry oscillations reduce the soil’s aggregate stability while simultaneously increasing soil respiration. However, in this zone, the microbial mechanisms of aggregate-scale soil respiration under water regime changes remain unclear.

This research sought to examine how aggregate-scale soil respiration is regulated by microbial community structure in response to changes in the water regime (i.e., from flooding to drying), with a specific focus on microbiota population, microbial co-occurrence tendencies, and their keystone taxa in networks. Specifically, this study investigates (1) how varying aggregate sizes influence soil respiration, bacterial and fungal community composition, and co-occurrence networks during flooding and drying; (2) how the microbial keystone taxa change during the flooding and drying periods and whether soil respiration is regulated by keystone taxa at the aggregate scale; and (3) how to reveal the regulatory mechanisms of aggregate-scale soil respiration for different flooding and drying treatments. Considering that soil respiration and microbial communities are susceptible to disturbance due to water regime changes, we hypothesized that (1) soil respiration rate and microbial community richness would decline with the decrease in soil aggregate size, and their respiration would increase with the shift from flooding to drying, and (2) the microbial keystone taxa would predominantly regulate soil respiration in both flooding and drying treatments.

## 2. Materials and methods

### 2.1. Experimental sites and soil sampling

**Figure 1:**
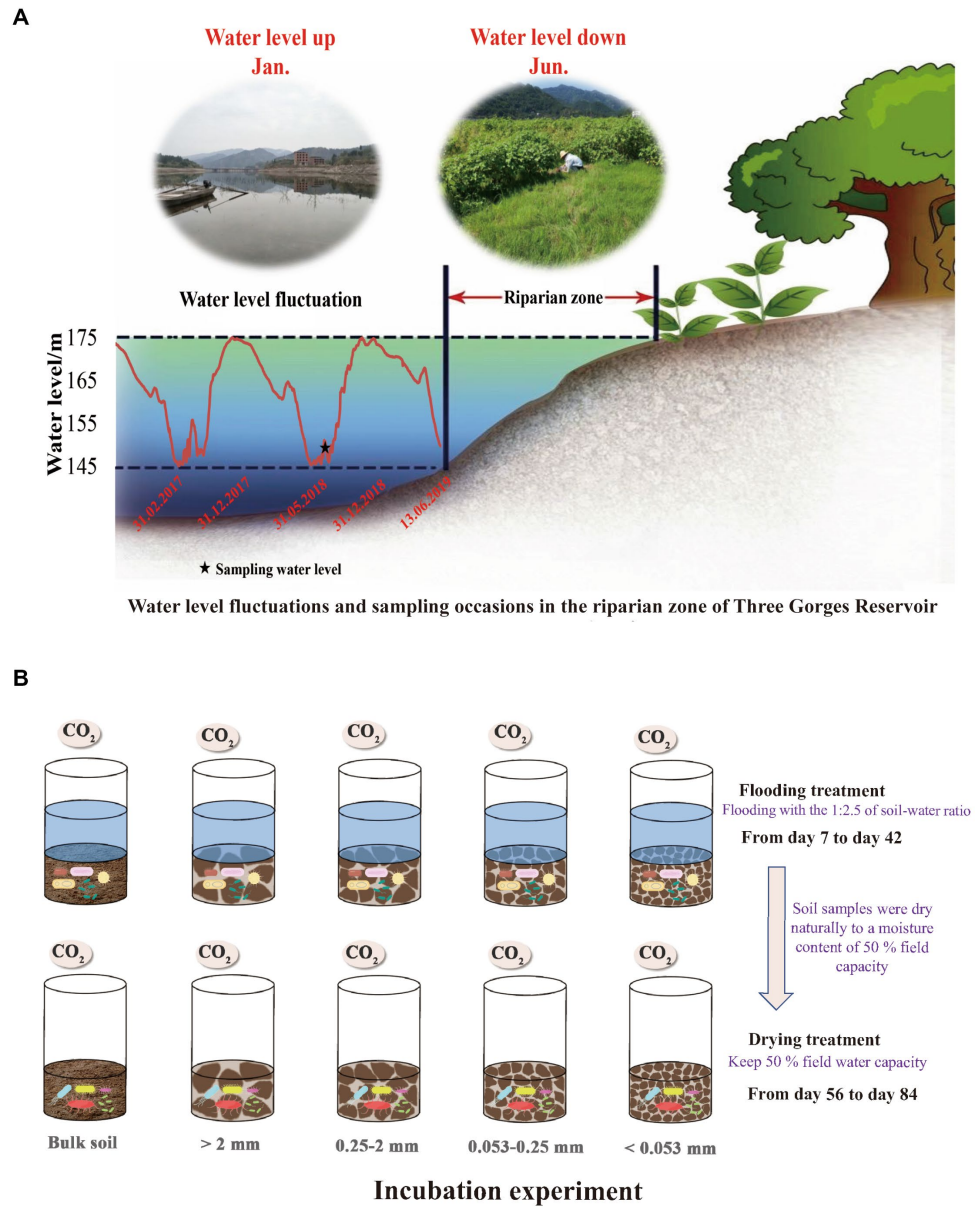
Water level changes in the Three Gorges Reservoir and sampling frequency in the riparian zone **(A)**; Schematic design and flow chart of the lab incubation experiment **(B)**.

In June 2018, soil samples for this research were taken from the Wuyangwan riparian zone (31°11′20″N, 108°27′40″E), which is representative of the riparian zones along the Pengxi River. As a result of the activities of the Three Gorges Dam, the water level of the Pengxi River, which is a secondary branch in the TGR of the Yangtze River, varies between 145 and 175 meters above sea level (m.a.s.l) in the summer and winter, respectively (Figure 1) since the TGR was fully impounded in 2010 (Zhu et al., 2020). With average annual temperatures of 18.2 ° C and average annual precipitation of 1,200 mm, the climate of this region may be described as a humid and mid-subtropical monsoon. The period from April through September, which is considered to be the plant growth season, receives over 60% of the total yearly precipitation (Zhu et al., 2020). Purple soil, formed from purple sandstone, is the dominant zonal soil type (Entisols in the World Reference Base) (Chen et al., 2014) with a texture dominated by silt loam (2.50% clay, 65.41% silt, and 30.09% sand) (Zhu et al., 2022b). Most native trees have died because of periodic flooding. Moreover, tillage is not allowed in the TGR riparian zone owing to environmental concerns. Thus, grasslands are the dominant land-use type, comprising flood-tolerant grasses such as *Xanthium sibiricum, Paspalum thunbergii*, and *Cynodon dactylon* (Ye et al., 2019). Since 2010, corn fields have been converted to selected grasslands, which were used as the study sites.

Based on an S-shaped sampling strategy and using a soil corer (5.7 cm diameter), nine soil samples, weighing approximately 1.5 kg were obtained at random from the grasslands from 0 to 10 cm depths. To avoid any impacts that might break the macroaggregates, we used a wooden mallet to carefully drive the corer into the soil.

### 2.2. Soil physicochemical properties

The soil that had been dried in an oven was used in the calculation of the soil bulk density (SBD). After mixing the sample with distilled water at a ratio of 1:2.5 soil to water, the pH of the soil was determined. SOC and TN were determined by the dry combustion method with a CN elemental analyzer (Vario Max CN Macro Elemental Analyzer, Elementar Analysensysteme GmbH, Hanau, Germany) (Nelson and Sommers, 1996).

### 2.3. Sieving of soil aggregates

Bulk soil (BS) samples were broken by hand along the fractures of the peds (> 8 mm). Plant residues, stones, visible fauna, and roots were excluded from the samples. Aggregate fractions were measured using wet sieving as described by Márquez et al. (2004). Briefly, a stack of sieves (2-, 0.25-and 0.053-mm) was used to manually fractionate air-dried samples of soil into four size classes in distilled water for 2 min at a rate of 30 times/min. The bulk soil was broken down into 4 aggregate fractions: > 2 mm (large macroaggregates, LM), 0.25–2 mm (small macroaggregates, SM), 0.053–0.25 mm (microaggregates, MI), and < 0.053 mm (silt and clay, SC). As soon as wet sieving was complete, the aggregates were given a gentle rinse in sterile water.

### 2.4. Incubation experiment

The prepared soils were introduced into 1,000 ml incubation jars (equivalent to 200 g of oven-dried soil in each), with three replicate jars of each treatment. In total, 15 glass jars were used for the incubation experiments, including three jars each for BS, LM, SM, MI, and SC. To accomplish soil microbial stability, we pre-incubated all soil samples at 15°C for 7 days with 50% water-filled pore space (Butterly et al., 2010; Jiang et al., 2021).

To simulate the soil moisture changes in the TGR riparian zone, the experiment was conducted at 15°C with two treatments: (a) flooding at a ratio of 1:2.5 soil to water and (b) drying with a moisture content of 50% field capacity. All incubated samples were placed in a constant-temperature incubator at 15°C, and in case it was deemed essential, deionized water was introduced into the mixture. Following the pre-incubation period, gas samples were withdrawn from the flooding incubation using a syringe on days 7, 14, 21, 28, 35, and 42. Subsequently, the flooding incubation experiment was terminated, and the soil samples were naturally dried to a moisture content of 50% field capacity on the 55th day. Gas samples were extracted on days 56, 63, 70, 77, and 84 of the drying incubation. Twice weekly, the jars were weighed, and water was replenished to prevent excessive evaporation and keep the humidity level consistent. Following the removal of the polyethylene film from the jars, their headspaces were purged with fresh air for approximately 15 min. Gas was collected in the jars after they were hermetically sealed with rubber septum covers. At 0 and 1 h after the jar had been sealed, a gas-tight syringe was used to extract about 30 ml of gas from the headspace. The levels of carbon dioxide in the samples were determined by the use of Gas chromatography (GC-2014, Shimadzu, Japan).

### 2.5. Microbial community analysis

Following the guidelines provided by the manufacturer of the MoBio PowerSoil DNA extraction kit, total DNA was extracted from 0.25 g of soil. Following amplification with fungal primers, ITS1F/ITS2R (Adams et al., 2013) and bacterial primers, 338\u00B0F/806R (Lee et al., 2012), samples were sent to the Novogene Biotechnology Co., Ltd. (Beijing, China) for sequencing via Illumina® MiSeq. To extract the valid data (clean data) after sequencing, the raw data were first demultiplexed and then subjected to quality filtering using the Trimmomatic program (Magoč and Salzberg, 2011). A table of operational taxonomic units (OTUs) was created by clustering the sequences. Using the UPARSE program (UPARSE v7.0.1001^1^; Edgar, 2013) Sequences from bacteria and fungi with a similarity of ≥97% were placed in the same OTU. BLAST was employed to search the UNITE (fungi) and RDP (bacteria) databases for matching sequences, and then representatives of each OTU were chosen for taxonomic annotation (Wang et al., 2007; Kõljalg et al., 2013). There were a total of 17,115 16S OTUs and 5,033 ITS OTUs discovered across all samples. Bacterial and fungal raw sequencing data were jointly submitted to the National Center for Biotechnology Information (NCBI) Sequence Read Archive (SRA) database under BioProject accession number PRJNA844584.

### 2.6. Calculations and statistical analysis

The soil microbial respiration was calculated according to the following formula (Wang et al., 2014):


(1) $$F=\rho \cdot \frac{v}{a}\cdot \frac{p}{p_{0}}\cdot \frac{t_{0}}{t}\cdot \frac{{\text{dC}}_{t}}{d_{t}}$$

Whereby, F denotes the CO_2_ flux (mg m^−2^ h^−1^); ρ (kg m^−3^) represents the CO_2_ density under normal circumstances; the effective volume is denoted by v (m^3^) while the bottom of the incubation jar is denoted by (m^2^); t_0_ represents the absolute temperature when circumstances are normalized; t signifies the absolute temperature within the jar; and $\frac{dC_{t}}{d_{t}}$ is the shift in the level of CO_2_ (m^3^ m^−3^) that occurred within the jar throughout the sampling duration (h).

R software (version 4.1.0) was used for every computation along with the data analyzes. The Levene’s and Kolmogorov–Smirnov tests were completed to correspondingly ensure the homogeneity of variances and the normality of the data before proceeding with the analysis. If the conditions were not met, a log or square-root transformation was applied. Soil aggregate fractions were separated into four groups, and their individual attributes were compared via analysis of variance (ANOVA). Soil respiration variations between aggregate fractions were analyzed through repeated-measure ANOVA. Also, an independent sample t-test was conducted to contrast the impacts of flooding and drying treatments on the aforementioned characteristics at the same aggregate scale. Two-way ANOVA was executed to determine the different moisture treatments, soil aggregate sizes, and their interactions with soil respiration.

Using the cmdscale function in the *vegan* package, principal coordinate analysis (PCoA) was utilized to investigate the differences in bacterial and fungal community architectures across the various treatments and aggregate fractions. To show the connections between microbial populations and to compute their topological features, we adopted the co-occurrence network inference (CoNet) in Gephi 0.9.2. Keystone species were identified as OTUs exhibiting a high degree, high eigenvector centrality, and high closeness/betweenness centrality (Wang et al., 2021). The highest average degree was considered a complex microbial network (Wagg et al., 2019). Soil respiration and keystone species abundance were also subjected to regression analyzes (Banerjee et al., 2016).

To determine how various predictor factors may be influencing soil respiration, partial least squares path modeling (PLS-PM) was carried out using the *plspm* program (Sanchez, 2013). Fourteen manifest variables [SOC, C/N ratio, pH, soil respiration, positive to negative edges (P/N) of the overall network (BPN), bacterial P/N related to keystone taxa (BKPN), bacterial average clustering coefficients (BACC), bacterial richness (BR), bacterial first dominant eigengenes BFDE, fungi richness (FR), fungal first dominant eigengenes (FFDE), fungal P/N of the overall network (FPN), fungal P/N related to keystone taxa (FKPN), and fungal average clustering coefficients (FACC)] and four latent variables (bacterial network, bacterial community composition, fungal network, and fungal community composition) were condensed for use in the PLS-PM. There were two or three manifest variables associated with each latent variable. The bacterial networks encompassed BPN, BKPN, and BACC. The bacterial community composition comprised BR and BFDE. Fungal networks included FR, FFDE, and FPN. The fungal community composition included FR and FFDE. We then computed the models’ path coefficients, which describe the direction and intensity of the linear correlations between the variables, as well as the explained variability (*R*^2^). Based on this information, we determined the overall influence (both direct and indirect) that each variable had on soil respiration. The path coefficients represent the direct impacts, while the indirect effects may be calculated by multiplying the direct effects by the indirect path’s path coefficients. The model’s overall prediction accuracy was measured by calculating its goodness of fit. Before running PLS-PM, we used a *car* package to ensure there wasn’t any multicollinearity between our chosen independent variables. In this investigation, we conducted regression random forest analysis with the *rfPermute* program to determine the most important soil variables for soil respiration. Variables with low levels of intercorrelation, a variance inflation factor of <10, significance levels of <0.05, and greater levels of mean squared error (MSE percent) were retained (Liaw and Wiener, 2002). Consequently, the proportion of soil aggregates, TN, and SBD was excluded (Supplementary Figure S1).

## 3. Results

### 3.1. Soil characteristics and microbial respiration

Flooding and drying influenced soil properties (Table 1). Flooding to drying generally decreased SOC and TN contents but slightly increased soil pH in all aggregate fractions. The SOC and TN contents of >2 mm aggregate fractions were substantially elevated relative to those of the other aggregate fractions in both the flooding and drying treatments. The SBD decreased as the aggregate size increased in both the flooding and drying treatments (i.e., LM < SM < MI < SC).

**Table 1:** Main soil properties under drying and drying treatments.

	SOC (g kg^−1^)		TN (g kg^−1^)		C:N (−)		pH		SBD		PSA
	Flooding	Drying	Flooding	Drying	Flooding	Drying	Flooding	Drying	Flooding	Drying	
BS	21.1bA	16.2bB	1.9aA	1.5bB	11.11bA	10.80bA	6.41aA	7.01aA	1.16abA	1.17abA	-
LM	27aA	24.2aA	1.6bA	1.6aA	16.88aA	15.13aA	6.53aA	6.92aA	1.07cA	1.09cA	38.97a
SM	17.3bA	16.3bA	1.4bA	1.3bA	12.36bA	12.54bA	6.75aA	6.92aA	1.13bA	1.13bA	30.56a
MI	12.1cA	10.4cA	1cA	0.9cA	12.10bA	11.56bA	6.88aA	6.99aA	1.18aA	1.19aA	14.33b
SC	12.1cA	11.7cA	1.3bA	1.2bA	9.31bA	9.75bA	6.94aA	6.79aA	1.21aA	1.23aA	16.33b

BS, bulk soil; LM, >2 mm aggregate fractions; SM, 0.25–2 mm aggregate fractions; MI, 0.053–0.25 mm aggregate fractions; SC, <0.053 mm aggregate fractions; SOC, soil organic carbon; TN, soil total nitrogen; C:N, soil carbon to nitrogen ratio; SBD, soil bulk density; PSA, percentage of the aggregate size fractions. Significant deviations among various aggregate size fractions within the same column are denoted by small letters; Significant variations (*p* < 0.05) between soil moisture treatments within the same row are denoted by capital letters.

**Figure 2:**
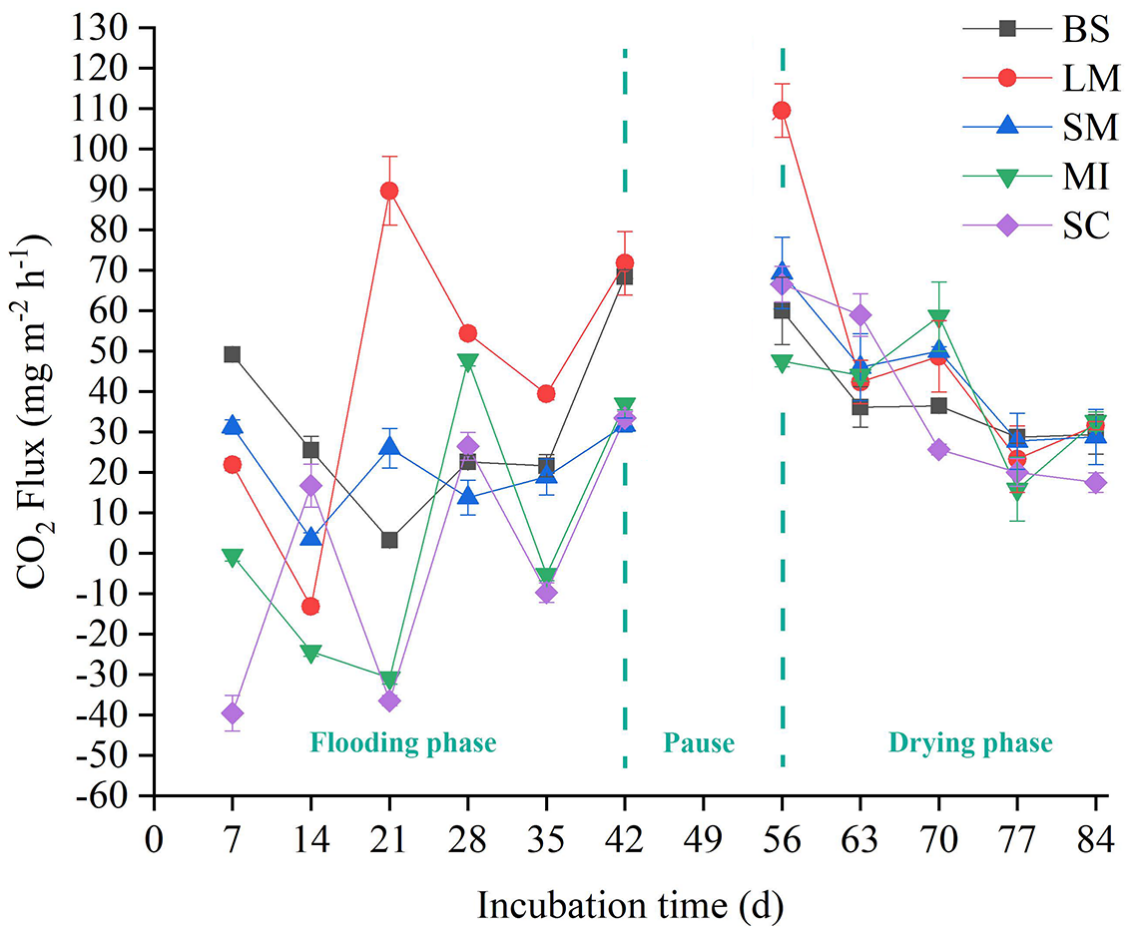
Flux of CO_2_ among soil aggregates in flooding and drying status. BS, bulk soil; LM, large macroaggregate, > 2 mm; SM, small macroaggregate, 0.25–2 mm; MI, microaggregate, 0.053–0.25 mm; SC, silt and clay, < 0.053 mm.

**Figure 3:**
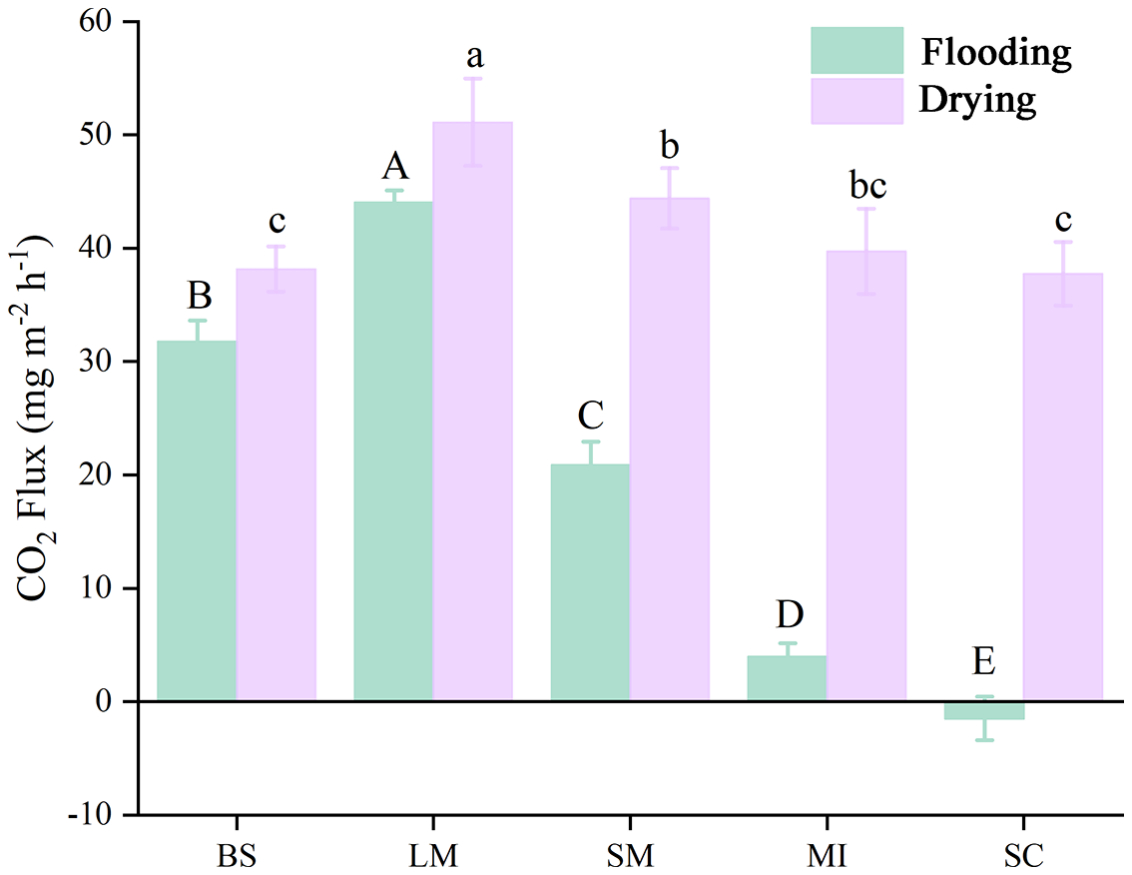
Mean soil respiration of different aggregate sizes in flooding and drying treatments during the incubation period. BS, bulk soil; LM, large macroaggregate, > 2 mm; SM, small macroaggregate, 0.25–2 mm; MI, microaggregate, 0.053–0.25 mm; SC, silt and clay, < 0.053 mm. A significant difference in soil respiration across aggregate sizes (*p* 0.05) in both the flooded and dried conditions is shown by the bars designated with capital letters and lowercase letters, correspondingly.

The soil CO_2_ flux (i.e., soil respiration) slightly increased with incubation time during the flooding phase (Figure 2). In contrast, soil respiration slightly decreased with increasing incubation time during the drying phase (Figure 2). Mean soil respiration dropped as the aggregate size became smaller in both the flooding and drying treatments (i.e., LM > SM > MI > SC). Notably, flooding to drying substantially enhanced the mean soil respiration (*p* < 0.05; Figure 3).

### 3.2. Abundance and composition of the microbial population

The soil microbial community richness is presented in Supplementary Table S1. The bacterial richness considerably decreased in response to the shift from flooding to drying. In contrast, the fungal richness substantially increased in response to the shift from flooding to drying. The maximum bacterial richness among the aggregate fractions in the flooding and drying conditions were MI (i.e., 5139.00 ± 38.73) and SC (i.e., 4290.67 ± 152.78), respectively. The minimum bacterial richness among the aggregate fractions in the flooding and drying conditions were SC (i.e., 4617.00 ± 107.41) and MI (i.e., 3671.33 ± 93.38), respectively. The maximum fungal richness among the aggregate fractions in the flooding and drying conditions were SM (i.e., 611.00 ± 22.15) and SC (i.e., 723.33 ± 86.33), respectively. The minimum fungal richness among the aggregate fractions in the flooding and drying conditions were MI (i.e., 512.67 ± 61.06) and SM (i.e., 663.67 ± 27.28), respectively. Nevertheless, no significant variance in fungal richness was discovered among the aggregate fractions under the drying treatment. Moreover, the shift from flooding to drying reduced the bacteria-to-fungi ratio in all the aggregate fractions.

**Figure 4:**
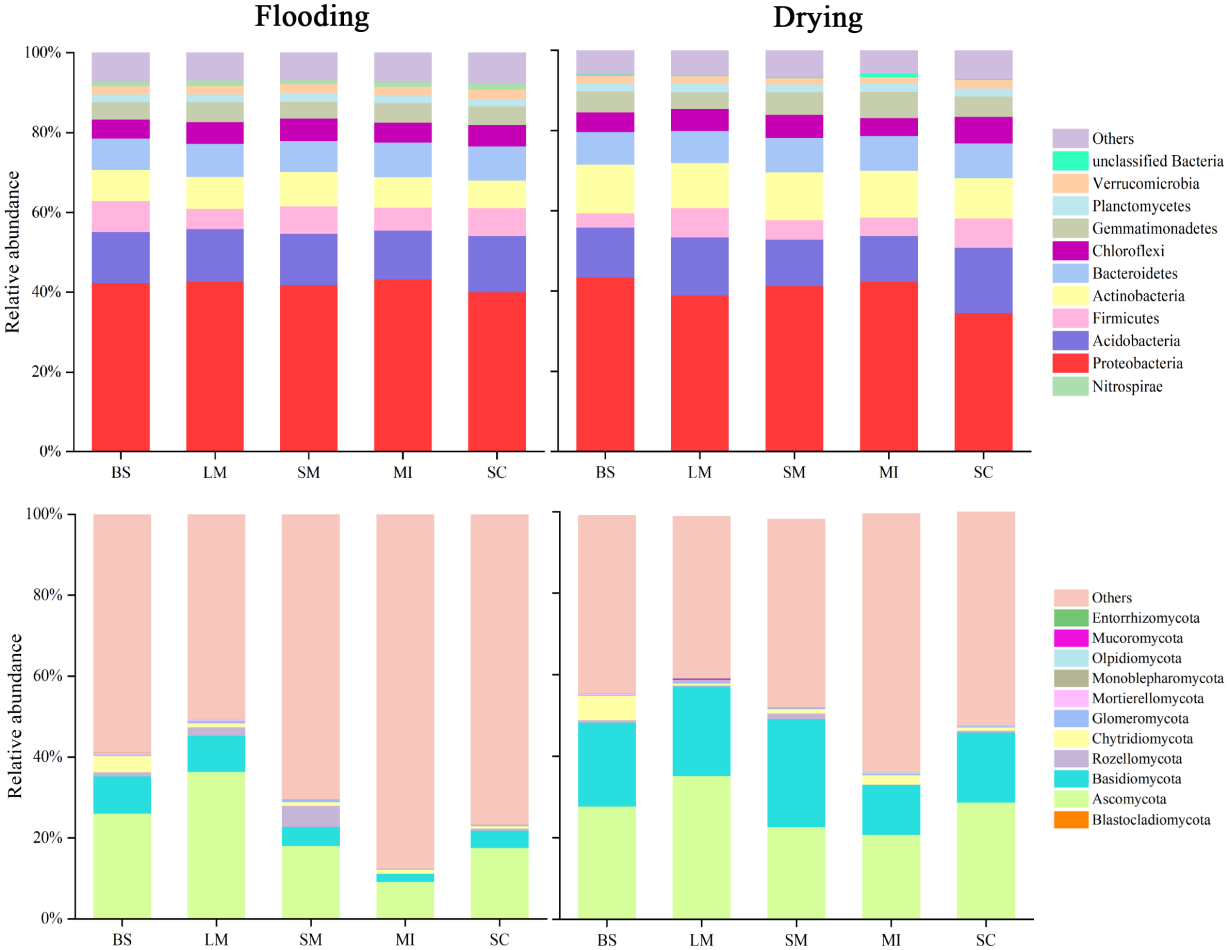
Aggregate size fractions during flooding and drying conditions and the relative abundance of key phyla within fungi and bacteria communities.

The relative abundance of the dominant bacterial and fungal phyla in soil aggregates under flooding and drying treatments are shown in Figure 4. The bacterial communities in soil aggregates predominately comprised *Proteobacteria* (34.6–43.3%), *Acidobacteria* (11.4–16.3%), *Actinobacteria* (6.8–12.1%), *Firmicutes* (3.6–7.7%), and *Planctomycetes* (1.9–2.2%). Compared with the flooding, the drying treatment resulted in an increase of 38.4–57.5% in the relative abundance of *actinobacteria*. The bacterial communities primarily included Ascomycota (9.3–36.5%), *Basidiomycota* (1.9–26.5%), *Rozellomycota* (0.1–5.2%), and *Chytridiomycota* (0.1–4.1%). Compared with the flooding treatment, the drying treatment generally enhanced the relative abundance of *Basidiomycota* and *Ascomycota* in soil aggregates.

**Figure 5:**
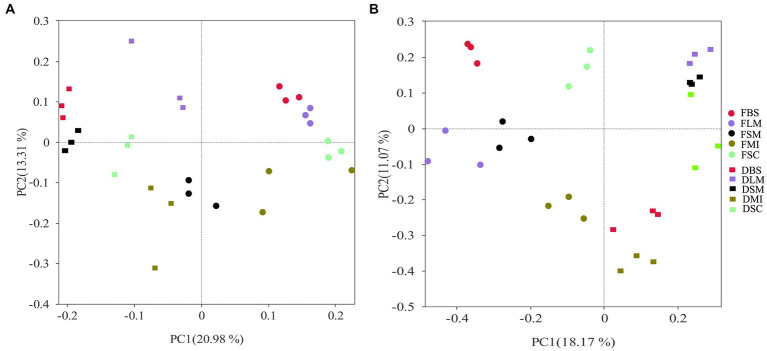
The impacts of moisture status and aggregate fractions on bacterial **(A)** and fungal **(B)** populations are shown using principal coordinate analysis predicated on the Bray-Curtis distances. Symbols of varying hues depict various aggregate fractions, each corresponding to a certain treatment of soil moisture. FBS, bulk soil in the flooding treatment; FLM, large macroaggregates in the flooding treatment; FSM, small macroaggregates in the flooding treatment; FMI, microaggregates in the flooding treatment; FSC, silt and clay in the flooding treatment; DBS, bulk soil in the drying treatment; DLM, large macroaggregates in the drying treatment; DSM, small macroaggregates in the drying treatment; DMI, microaggregates in the drying treatment; DSC, silt, and clay in the drying treatment.

The PCoA revealed that both bacterial and fungal community compositions were distinct among the aggregate fractions and treatments (Figure 5). Three samples from the same moisture conditions were grouped, regardless of the aggregate size. Moreover, the samples from different moisture conditions were completely separated from each other. The first two principal coordinates together explained 34.29 and 29.24% of the differences in soil bacteria and fungi community compositions, correspondingly.

### 3.3. Soil microbial co-occurrence networks

**Figure 6:**
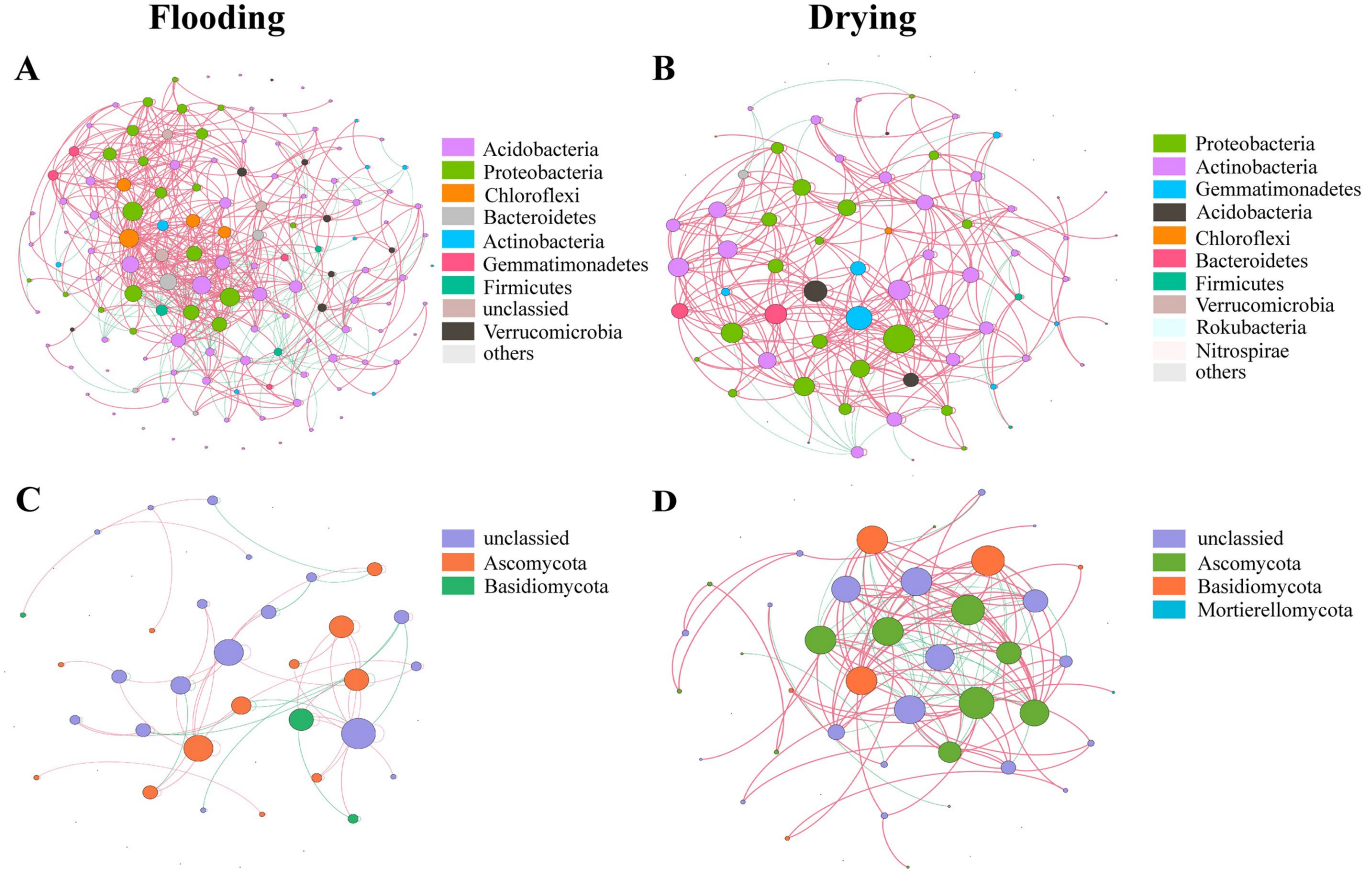
Networks showing the co-occurrence of bacterial **(A,B)** and fungal **(C,D)** populations under flooding and drying, respectively. A connection represents a statistically significant (*p* < 0.005) relationship between two OTUs. In a network, a node’s size is equivalent to the number of its connections (i.e., degree), and the Pearson correlation coefficients are used to determine the thickness of the edges connecting the nodes. Positive and negative interactions are denoted by the red and green edges, correspondingly.

To evaluate differences in network features between individual treatments and for every aggregate fraction, we generated 20 subnetworks from the bacterial and fungal populations. Supplementary Table S3 displays the results of the ANOVA. Both bacteria and fungi community compositions exhibited modified co-occurrence patterns in response to the varying soil moisture treatments (Figure 6). The changes in the soil moisture conditions substantially affected the dynamics of the microbial and fungal populations’ interplay. The shift from flooding to drying increased the ratio of positive to negative edges (P/N), average degree, and average clustering coefficient in all bacteria and fungi networks and most subnetworks (Table 2; Supplementary Tables S2, S3).

**Table 2:** Topological properties of bacterial and fungal subnetworks.

	Bulk/Aggregate soil	Treatments	P/N of the whole network	P/N associated with keystone taxa	Average degree	Average clustering coefficient
Bacterial networks	Bulk soil	Flooding	1.891	9.000	2.220	0.234
		Drying	2.616	4.000	3.359	0.301
	>2 mm	Flooding	0.127	0.095	2.987	0.213
		Drying	1.459	0.125	3.488	0.322
	0.25–2 mm	Flooding	0.134	0.167	2.920	0.224
		Drying	1.462	3.000	4.014	0.276
	0.053–0.25 mm	Flooding	0.186	0.170	3.593	0.247
		Drying	1.531	1.750	3.754	0.300
	<0.053 mm	Flooding	2.262	4.167	3.618	0.324
		Drying	3.718	13.500	5.531	0.320
Fungal networks	Bulk soil	Flooding	0.495	0.100	8.179	0.288
		Drying	0.300	15.000	5.337	0.264
	>2 mm	Flooding	0.530	1.375	6.248	0.342
		Drying	0.712	15.500	7.538	0.381
	0.25–2 mm	Flooding	0.722	0.444	4.656	0.303
		Drying	0.793	3.714	8.803	0.336
	0.053–0.25 mm	Flooding	0.526	1.500	4.360	0.258
		Drying	0.713	2.000	5.930	0.299
	<0.053 mm	Flooding	0.946	13.000	8.041	0.335
		Drying	1.002	1.000	10.271	0.362

The changes in soil moisture conditions changed the keystone taxa (Table 3). For bacterial networks, *Acidobacteria* (*unclassified Acidobacteria* at the order level), *Acidobacteria* (*Acidobacteriales*), *Gemmatimonadetes* (*Gemmatimonadales*), *Chloroflexi* (*Anaerolineales*), and *Bacteroidetes* (*Cytophagales*) were the keystone taxa in the flooding treatment, whereas, in the drying treatment, the keystone taxa were *Proteobacteria* (unclassified), *Proteobacteria* (*Rhizobiales*), *Gemmatimonadetes* (*Gemmatimonadales*), *Proteobacteria* (*Sphingomonadales*), and *Actinobacteria* (*Solirubrobacterales*). For fungal networks, the keystone taxa in the flooding and drying treatments were *Ascomycota* (*Hypocreales*) and *Basidiomycota* (*Agaricales*), respectively.

**Table 3:** The keystone taxa in the bacterial and fungal networks in flooding and drying treatments.

ID	Phylum	Order	Degree	Eigen centrality	Closeness centrality	Betweeness centrality	Treatment
OTU326	*Acidobacteria*	*Unclassified*	32	0.97	0.50	621.44	
OTU110	*Acidobacteria*	*Acidobacteriales*	37	1	0.38	448.98	
OTU88	*Proteobacteria*	*Unclassified*	31	0.96	0.40	215.48	Flooding
OTU1276	*Chloroflexi*	*Anaerolineales*	37	0.92	0.38	525.64	
OTU714	*Bacteroidetes*	*Cytophagales*	32	0.74	0.38	411.07	
OTU89	*Gemmatimonadetes*	*Gemmatimonadales*	36	1	0.42	353.45	
OTU162	*Proteobacteria*	*Rhizobiales*	34	0.98	0.41	386.78	
OTU88	*Proteobacteria*	*Unclassified*	32	0.96	0.41	341.53	Drying
OTU13562	*Proteobacteria*	*Sphingomonadales*	37	0.91	0.42	386.78	
OTU144	*Actinobacteria*	*Solirubrobacterales*	34	0.90	0.40	370.63	
OTU13	*Unclassified*	*NA*	9	1	0.71	37	
OTU12	*Ascomycota*	*Hypocreales*	8	0.73	0.57	16	
OTU21	*Ascomycota*	*NA*	8	0.72	0.63	31.5	Flooding
OTU2732	*Ascomycota*	*Hypocreales*	7	0.75	0.38	15	
OTU15	*Unclassified*	*Unclassified*	7	0.80	0.57	7	
OTU12	*Ascomycota*	*Hypocreales*	17	0.92	0.49	134.65	
OTU11	*Basidiomycota*	*Agaricales*	16	1	0.48	16.07	
OTU222	*Basidiomycota*	*Agaricales*	15	0.96	0.52	121.19	Drying
OTU3	*Basidiomycota*	*Agaricales*	15	0.99	0.47	41.81	
OTU477	*Unclassified*	*Unclassified*	15	0.89	0.47	786.78	

Variation in the keystone taxa was observed across soil moisture manipulations (Supplementary Table S3). Bacterial subnetworks showed changes in their keystone taxa in response to manipulations of soil moisture and particle size distributions. P/N was solely influenced by soil moisture treatments for fungal subnetworks, whereas the average clustering coefficient was influenced by the interplay of aggregate sizes and soil moisture treatments.

### 3.4. Keystone taxa regulated soil respiration

**Figure 7:**
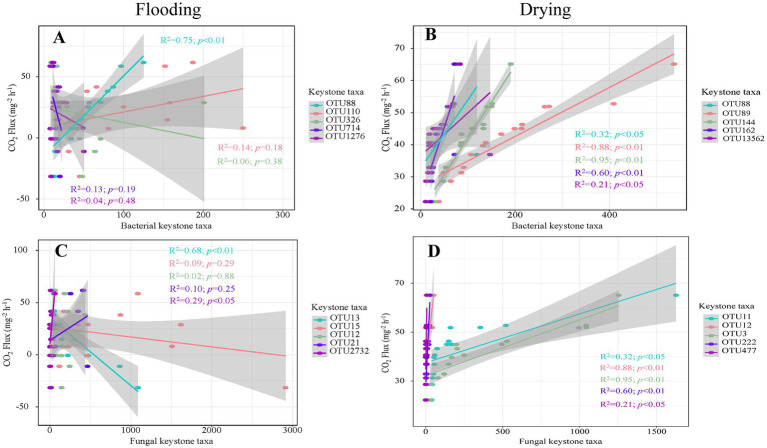
The relationships of soil respiration with the richness of key bacterial **(A,C)** and fungal members **(B,D)**.

The keystone taxa abundance was shown to have a strong correlation with soil respiration in regression analyzes (Figure 7). For bacteria, soil respiration was positively influenced by OTU88 (*Proteobacteria*; *R*^2^ = 0.75, *p* < 0.01) under flooding treatment. Under drying treatment, soil respiration was positively influenced by OTU88 (i.e., *Proteobacteria*; *R*^2^ = 0.32, *p* < 0.01), OTU89 (*Gemmatimonadetes*; *R*^2^ = 0.88, *p* < 0.01), OUT144 (*Actinobacteria*; *R*^2^ = 0.95, *p* < 0.01), OTU162 (*Actinobacteria*; *R*^2^ = 0.60, *p* < 0.01), and OTU13562 (*Actinobacteria*; *R*^2^ = 0.21, *p* < 0.05). For fungi, soil respiration was positively influenced by OTU2732 (*Ascomycota*; *R*^2^ = 0.29, *p* < 0.05) under flooding treatment. Under flooding treatment, soil respiration was negatively influenced by OTU13 (*unclassified fungus*; *R*^2^ = 0.68, *p* < 0.01). Moreover, under drying treatment soil respiration was positively influenced by OTU11 (*Basidiomycota*; *R*^2^ = 0.32, *p* < 0.01), OTU12 (*Ascomycota*; *R*^2^ = 0.88, *p* < 0.01), OTU3 (*Basidiomycota*; *R*^2^ = 0.95, *p* < 0.01), OTU222 (*Basidiomycota*; *R*^2^ = 0.60, *p* < 0.01), and OTU477 (*unclassified fungus*; *R*^2^ = 0.21, *p* < 0.05).

### 3.5. The influence of soil characteristics and microbial community on soil respiration

A regression random forest analysis was showed that the key soil parameters that were linked to soil respiration were SOC, C/N ratio, and pH (Supplementary Figure S1). Soil respiration was also significantly influenced by the make-up of the microbial population (as measured by the first dominant eigengenes, FDE) and the features of the microbial network (as measured by the P/N ratio related to keystone taxa and the mean cluster coefficients).

**Figure 8:**
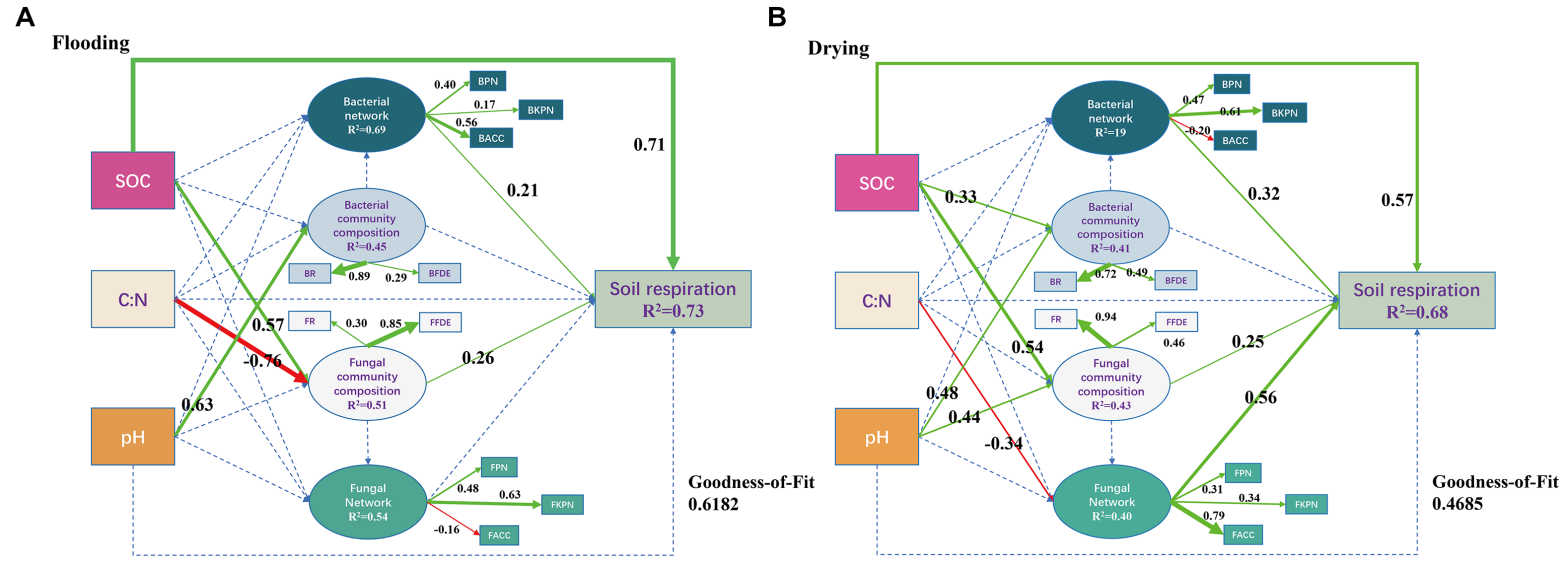
Soil respiration derived from different sizes of aggregates after exposure to flooding **(A)** and drying **(B)** treatments were modeled using a partial least squares path model of the impacts of soil characteristics, microbial population structure, and microbial network topological parameters **(B)**. Positive paths (*p* < 0.05) are denoted by solid arrows, while insignificant paths (*p* > 0.05) are illustrated by the dotted arrows. The goodness-of-fit (GoF) statistic was employed to evaluate the model. SOC, soil organic carbon; C:N, the ratios of total carbon and nitrogen; BPN, P/N of the entire network; BKPN, P/N of the bacterial network related to keystone taxa; BACC, average clustering coefficients of the whole network; BR, the relative abundance of bacteria; BFDE, first dominant eigengenes of the bacterial community composition; FPN, P/N of the entire network; FKPN, P/N of the bacterial network related to keystone taxa; FACC, average clustering coefficients of the fungal network; FR, the relative abundance of fungi; FFDE, first dominant eigengenes of fungal community composition.

In both the flooded and dried soils, PLS-PM analysis showed causative links between soil respiration, soil microbial network, soil microbial community composition, and environmental factors (Figure 8). In the flooding treatment, SOC, bacterial network, and fungal community composition exhibited a remarkable direct effect on soil respiration, with path coefficients of 0.71, 0.21, and 0.26, correspondingly (*p* < 0.05). Additionally, the SOC and C/N ratios both exhibited substantial indirect effects on soil respiration through fungal community composition, with path coefficients of 0.15 and-0.20, correspondingly (*p* < 0.05). Consequently, SOC had a strong beneficial effect on soil respiration, as measured by a path coefficient of 0.86. Bacterial networks predominantly and significantly relied on BPN and BACC with loading coefficients of 0.40 and 0.56, correspondingly (*p* < 0.05). Fungal community composition dominantly relied on their FFDE with a loading coefficient of 0.85 (*p* < 0.05).

In the drying treatment, SOC, bacterial network, fungal community composition, and the fungal network exhibited substantial direct effects on soil respiration, as indicated by path coefficients of 0.57, 0.32, 0.25, and 0.56, correspondingly (*p* < 0.05). Moreover, the SOC and pH exhibited substantial indirect effects on soil respiration through fungal community composition, as illustrated by coefficients of 0.14 and 0.11, correspondingly (*p* < 0.05). By way of the microbial community, the C/N ratio strongly influenced soil respiration (path coefficient = −0.19) (*p* < 0.05). Furthermore, SOC had a path coefficient of 0.71, indicating that it considerably and positively influenced soil respiration. The bacterial network dominantly and significantly relied on BKPN with a loading coefficient of 0.61 (*p* < 0.05). Moreover, fungal community composition predominantly relied on FR with a loading coefficient of 0.94 (*p* < 0.05). The fungal network predominantly relied on FACC with a loading coefficient of 0.79 (*p* < 0.05).

## 4. Discussion

### 4.1. Soil respiration impacted by flooding and drying conditions at the aggregate scale

Soil respiration reflects short-term dynamics of SOC, which are primarily affected by several biotic and abiotic factors, like microorganisms, substrate availability, and water content (Luo and Zhou, 2006; Wang et al., 2020). In this study, the respiration of different-sized soil aggregates slightly increased with the incubation time during the flooding phase, whereas it slightly decreased with incubation time during the drying phase (Figure 2). These differences may be caused by the discrepancy between the labile and recalcitrant carbon fractions used by microbes (Rusalimova and Barsukov, 2006). Generally, labile organic carbon is preferentially decomposed by soil microorganisms during the early stages of incubation. However, microorganisms began to use the recalcitrant organic carbon fraction which is more difficult to decompose in the late stage of incubation (Hao et al., 2008; Rabbi et al., 2014; Bimüller et al., 2016). In addition, the soil respiration rate fluctuated during the incubation process, which was likely because of soil disturbance caused by the pretreatment process, such as the collection of soil samples (Hao et al., 2008).

The variations in soil respiration at different aggregate sizes reflect the dominant roles of carbon and nitrogen contents and microbial activity within the aggregates under relatively uniform incubation conditions (Gupta and Germida, 1988; Kan et al., 2020). Our results showed that significant differences in the respiration of different size fractions of soil aggregates were observed under the same incubation conditions, and the mean soil respiration rate generally increased with the soil aggregate size-classes. These results support our first hypothesis, conforming to the conclusions drawn by Noellemeyer et al. (2008). The reasons for this are as follows. First, in the absence of plant roots, soil respiration is primarily derived from the decomposition of organic carbon by soil microorganisms (Ontl and Schulte, 2012). Further, the activities of microorganisms responded to the discrepancy in the amount and stability of organic carbon in different soil aggregate sizes, thereby leading to variations in soil respiration among different aggregate size-classes (Trivedi et al., 2017). Second, macroaggregate-associated carbon primarily derives from labile organic carbon (e.g., fresh plant residues) that easily decomposes, whereas microaggregate-associated carbon primarily comprises humus that is difficult to decompose and use (Six et al., 1999). Moreover, because the pore necks in microaggregates are <0.2 μm in width, the pores in these structures are impenetrable to bacteria, thereby inhibiting biological activity (Erktan et al., 2020; Zhu et al., 2022b).

The mean soil respiration of all aggregate size fractions in the drying treatment was substantially elevated in contrast to the flooding treatment under a constant temperature (Figure 3). The results primarily showed the inhibition of organic matter decomposition, as soil pore spaces are occupied by water in the flooding treatment (Gorham, 1995); Additionally, during the drying treatment, 50% field water retention capacity was maintained in the soil samples since it is suitable for microbial activity, particularly in the early stage of measurement (Manzoni et al., 2012).

### 4.2. Microorganisms in soil aggregates under flooding and drying treatments

Soil aggregates act as heterogeneous microhabitats for highly spatially organized microorganism communities (Upton et al., 2019). In this study, the maximum bacterial abundances among the aggregate fractions under flooding and drying conditions were MI and SC, respectively. SM had the maximum fungal abundance under flooded conditions. These findings were comparable to those obtained in earlier investigations which revealed that the bacterial and fungal abundances are maximum in <0.25 mm and > 0.25 mm aggregates, correspondingly (Zhang et al., 2014; Bach et al., 2018; Wilpiszeski et al., 2019). The presence of protected habitats in microaggregates may facilitate niche creation for bacteria by excluding their predators (protozoa) and competing with fungi, since predation serves as a crucial structuring force for bacterial communities (Zhang et al., 2014). Additionally, due to the narrow gaps that exist between microaggregates, silt, and clay fractions, it is physically impossible for fungi to penetrate the interior of these habitats (Zhang et al., 2014; Erktan et al., 2020).

Soil water regimes, which are associated with most soil properties and processes, have a profound influence on the structure and function of the soil microbial population in the soil (Brockett et al., 2012; Schimel, 2018). In this study, the shift from flooding to drying decreased the bacteria–fungi ratio in all aggregate size fractions. Notably, fungi are heterotrophic and essentially aerobic with limited anaerobic capabilities (McGinnis and Tyring, 1996). The shift from flooding to drying changed the soil microenvironment and improved the exchange of air and heat, which was conducive to soil fungal aeration. Fungal communities are generally more resistant to environmental alterations than bacterial communities (Lin et al., 2019). Consequently, fungi rapidly reproduce, thereby improving their diversity (Zhang, 2010). In addition, soil bacteria might adjust their expression of certain metabolic processes (e.g., resulting in the synthesis of compounds with osmoprotective properties) in response to the stress environment and maintain their growth during flooding (Chowdhury et al., 2019). For bacteria, *Proteobacteria* were the dominant taxa in both flooding and drying treatments. *Acidobacteria* and *Bacteroidetes* were the subdominant groups in the flooding and drying treatments, respectively. Among fungi, the dominant taxa were *Ascomycetes* and *Basidiomycetes*. However, the shift from flooding to drying considerably increased the relative abundance of Basidiomycetes. These results were in line with those found in earlier investigations, which revealed that the microbial communities *Proteobacteria*, *Acidobacteria*, *Bacteroidetes*, *Ascomycetes*, and *Basidiomycetes* are abundant in wetland soils which are frequently anaerobic (Tait et al., 2007; Peralta et al., 2013). Moreover, the relative abundance of dominant microbial taxa generally decreased with an increase in aggregate size, which might be related to the nutrient strategy of the microbial community (Fierer et al., 2007; Zhang et al., 2016) where macroaggregates tend to be nutrient-rich, whereas microaggregates are relatively barren (Fonte et al., 2014).

The co-occurrence patterns of soil microbes could be revealed by using network analysis, which is conducive to acquiring a more comprehensive knowledge of the structure of microbial communities and the biological principles that guide community formation (Barberán et al., 2012). Herein, in both the flooding and drying conditions, *Proteobacteria* and *Actinobacteria* predominated in the bacterial network, while *Ascomycota* predominated in the fungus network, indicating that these phyla could be the most influential in determining the overall architecture of microbiomes. This is likely because *Proteobacteria*, *Actinobacteria*, and *Ascomycota* can resist external pressure and disturbances (Zhou et al., 2020; Faitová, 2021). This finding aligns with the conclusions drawn by Ye et al. (2021) in their study of co-occurrence patterns in the soil microbial network within the riparian zone of the TGR. In addition, the fungal network exhibited higher average degrees, and more nodes and edges in the drying treatment than in the flooding treatment, suggesting that the shift from flooding to drying enhanced the connectivity and complexity of the fungal network (Wagg et al., 2019; Ye et al., 2021). These results are in agreement with those of earlier research showing that flooding drastically decreases the complexity of co-occurrence networks (Zhang et al., 2019; Gao et al., 2021) owing to resource limitations and environmental stresses, such as severe flooding stress, reduced water, and availability of nutrients (Malik et al., 2020; Qiu et al., 2021). In addition, positive linkages in the bacterial network, especially those linked to keystone taxa, rose as flooding duration reduced, whereas negative ones dropped as a result of the shift from flooding to drying (i.e., the P/N of the overall network and that related to keystone taxa) (Table 2). This indicates that the shift from flooding to drying increased niche breadths by eliminating flooding stress and reactivating aerobic microbes to enhance the availability of organic materials (Malik et al., 2020), thereby mitigating competition (Banerjee et al., 2016) and exhibiting favorable co-occurrence patterns with selected copiotrophic keystone taxa (Wang et al., 2021).

### 4.3. Soil respiration regulated by keystone taxa at the aggregate scale

Soil respiration is determined by the richness of certain taxa (Banerjee et al., 2016). Confirming our second hypothesis, both bacterial and fungal keystone taxa were found using the network analysis, and they were shown to have substantial ties to soil respiration (Figure 7). The abundance of *Proteobacteria* in bacteria and *Ascomycota* (*Hypocreales*) in fungi exhibited remarkably favorable impact on soil respiration during flooding treatment. *Proteobacteria*, *Proteobacteria* (*Rhizobiales*), *Gemmatimonadetes* (*Gemmatimonadales*), *Proteobacteria* (*Sphingomonadales*), and *Actinobacteria* (*Solirubrobacterales*) in bacteria and *Ascomycota* (*Hypocreales*), *Basidiomycota* (*Agaricales*), an unclassified taxon, in fungi, exhibited strong positive effects on soil respiration in the drying treatment. *Proteobacteria*, *Firmicutes*, and *Actinobacteria* have been found to decompose plant polymers by releasing soil enzymes like xylanases and β-glucosidase (Wang et al., 2010; Tiwari et al., 2016; Zheng et al., 2018). Earlier literature has shown that the families of α-*Proteobacteria* are decomposers of both fresh and soil organic matter (Bernard et al., 2012; Liu et al., 2021). Herein, both *Rhizobiales* and *Sphingomonadales* belong to *α*-*Proteobacteria*, which positively affected soil respiration. Actinobacteria are an essential bacterium class that participates in many activities throughout ecosystems, including the breakdown of organic molecules, which can mineralize fused aromatic C-ring structures (Faitová, 2021). *Gemmatimonadetes* are typically abundant and active in low-moisture soils, playing a vital role in driving soil carbon cycling processes (He et al., 2020; Fan et al., 2021). Thus, our findings illustrated that *Gemmatimonadetes* positively affected soil respiration during the drying treatment rather than during the flooding treatment. In addition, an unclassified keystone fungus exhibited a considerable negative impact on soil respiration in the flooding treatment, which could explain why negative rates of soil respiration were detected during the drying treatment. *Agaricales*, an order of *Basidiomycota*, is an extremely common soft-rot fungus that aids in decomposing dead wood and litter and may generate a spectrum of hydrolytic enzymes capable of breaking down humic and lignin acids (Voříšková and Baldrian, 2013). During decomposition, the prevalent and persistent ascomycetous fungi (*Hypocreales*) are the endophytes of a wide variety of plants and also include extracellular fungi that produce enzymes (Voříšková and Baldrian, 2013; Herzog et al., 2019). However, the keystone taxa *Rhizobiales*, *Gemmatimonadales*, *Sphingomonadales*, and *Solirubrobacterales*, *Hypocreales*, *Agaricales* are uncultured bacterial or fungal orders. Therefore, further studies are required to determine how they affect the composition and function of soil microbes (Wang et al., 2021). Additionally, keystone taxa should be selectively excluded in future studies to verify the changes in species interactions affecting soil respiration (Zheng et al., 2021).

### 4.4. Regulating mechanisms of soil respiration at the aggregate scale in flooding and drying treatments

Microbial community structure and network properties assume critical roles in the dynamics of SOC (Banerjee et al., 2016; Zheng et al., 2021). In both flooding and drying treatments, aggregate fractions remarkably modified the composition of microbial communities and microbial networks, primarily by altering their associated SOC levels (Cookson et al., 2005). In addition, SOC was the dominant regulator of soil respiration, with a path coefficient of 0.86 in the flooding treatment and 0.71 in the drying treatment (Figure 8), suggesting that substrate supply was the major factor affecting CO_2_ release in this study (Luo and Zhou, 2006). Furthermore, the mechanisms regulating soil respiration were distinct between the flooding and drying treatments (Figure 8).

In the flooding treatment, except for SOC, soil respiration was principally modulated by the ACC and P/N of the entire network and FFDE. However, in the drying treatment, soil respiration was predominantly modulated by FR, FACC, and P/N associated with the bacterial keystone taxa. These results show that the shift from flooding to drying changed the regulatory mechanisms of soil respiration, particularly the fungal network. Archived literatures illustrates that decreasing competitive interactions with keystone taxa enhances soil respiration (Wang et al., 2021), whereas soil respiration diminishes when competitive interactions increase among keystone taxa (Chen et al., 2019). The shift from flooding to drying relieved water stress, thereby alleviating interactions that are competitive with the co-occurrence networks’ keystone taxa. Moreover, C mineralization is less energy-efficient during anaerobic degradation (Wang et al., 2021). Therefore, the shift from flooding to drying increased the average soil respiration rate (Figure 3). Notably, the relieved water stress increases oxygen availability, which creates copiotrophic environments for fungi that prefer aerobic conditions (McGinnis and Tyring, 1996) and interacts with other species by using both labile and recalcitrant carbon fractions (Bian et al., 2022). Also, the effect of the bacterial network on soil respiration increased from a path coefficient of 0.21 to 0.32 (Figure 8), which revealed that the dominant bacterial network with positive interaction in aerobic conditions facilitated the utilization of carbon sources by microorganisms, thereby stimulating soil respiration (Wang et al., 2021).

## 5. Conclusion

Our study reveals that the shift from flooding to drying changes the microbial community composition and keystone taxa, thereby enhancing the microbial respiration of soil aggregates. Specifically, soil respiration decreases with a decrease in aggregate size in both flooding and drying treatments. Additionally, the microbial respiration of soil aggregates is substantially higher in the drying treatments than in the flooding treatment as a result of the changes in keystone taxa. Notably, the fungal community composition and network properties dominate the changes in microbial respiration of soil aggregates during the flooding to the drying process. This study reveals the crucial roles of fungal community composition and co-occurrence network properties in regulating soil respiration during the shift from flooding to drying conditions. Moreover, this analysis offers valuable knowledge of the mechanisms of soil respiration changes at the aggregate scale under different water regimes.

## Data availability statement

The original contributions presented in the study are included in the article/Supplementary material, further inquiries can be directed to the corresponding authors.

## Author contributions

KZ: conceptualization, methodology, validation, formal analysis, investigation, writing–original draft, visualization, and funding acquisition. WJ: writing–review and editing. YM: investigation and data curation. SW: methodology and funding acquisition. PH: conceptualization, resources, writing–review and editing, supervision, and project administration. All authors contributed to the article and approved the submitted version.

## Funding

This study was sponsored by Natural Science Foundation of Chongqing, China (2022NSCQ-MSX1111), “Through Train” for Doctors in Chongqing (sl202100000124), the National Natural Science Foundation of China (41771266), the Three Gorges’ follow-up scientific research project from Chongqing Municipal Bureau of Water Resources (No. 5000002021BF40001). PH is also supported by the “Light of West China” Program funded by the Chinese Academy of Sciences. China Postdoctoral Science Foundation (2021M703137), Chongqing Postdoctoral Science Foundation (cstc2021jcyj-bsh0080).

## Conflict of interest

The authors declare that the research was conducted in the absence of any commercial or financial relationships that could be construed as a potential conflict of interest.

## Publisher’s note

All claims expressed in this article are solely those of the authors and do not necessarily represent those of their affiliated organizations, or those of the publisher, the editors and the reviewers. Any product that may be evaluated in this article, or claim that may be made by its manufacturer, is not guaranteed or endorsed by the publisher.

## Footnotes

^1^ http://drive5.com/uparse
