# Supplementary material for: Shift from flooding to drying enhances the respiration of soil aggregates by changing microbial community composition and keystone taxa
Source: Front Microbiol. 2023 May 12;14:1167353. doi: 10.3389/fmicb.2023.1167353 (PMC10214030; doi:10.3389/fmicb.2023.1167353)
Supplement: Supplementary file 1 [file Data_Sheet_1.docx]

Supplementary Data

**Manuscript Title:**

**Shift from flooding to drying enhances the respiration of soil aggregates by changing microbial community composition and keystone taxa**

Kai Zhu, Weitao Jia, Yu Mei, Shengjun Wu, Ping Huang *

Key Laboratory of Reservoir Aquatic Environment, Chongqing Institute of Green and Intelligent Technology, Chinese Academy of Sciences, Chongqing 400714, China.

|  | Bacterial richness | | Fungal richness | | Bacteria/fungi ratio | |
| --- | --- | --- | --- | --- | --- | --- |
|  | Flooding | Drying | Flooding | Drying | Flooding | Drying |
| BS | 5065.00 ± 280.01bA | 3874.67 ± 330.74cB | 592.33 ± 15.33abB | 690.00 ± 34.05aA | 8.55 | 5.62 |
| LM | 5024.00 ±29.96bA | 4049.33 ± 233.94bB | 543.67 ± 44.77abB | 679.67 ± 72.79aA | 9.24 | 5.96 |
| SM | 5091.67 ±222.26bA | 3981.00 ± 61.08bcB | 611.00 ± 22.15aB | 663.67 ± 27.28aA | 8.33 | 5.99 |
| MI | 5139.00 ± 38.73aA | 3671.33 ± 93.38cB | 512.67 ± 61.06bB | 685.33 ± 67.30aA | 10.02 | 5.36 |
| SC | 4617.00 ± 107.41cA | 4290.67 ± 152.78aB | 565.33 ± 70.55abB | 723.33 ± 66.33aA | 8.17 | 5.93 |

**Table S1** Bacterial and fungal richness in different aggregate sizes.

Notes: BS, bulk soil; LM, >2 mm aggregate fractions; SM, 0.25-2 mm aggregate fractions; MI, 0.053-0.25 mm aggregate fractions; SC, <0.053 mm aggregate fractions; Different small letters in the same column indicate significant differences among different aggregate fractions, different capital letter case in the same row indicate significant differences between different water moisture treatments (*p* <0.05).

**Table S2** Topological properties of bacterial and fungal networks.

| Network topological properties | Bacteria | | Fungi | |
| --- | --- | --- | --- | --- |
|  | Flooding | Drying | Flooding | Drying |
| No. of Nodes | 134 | 81 | 48 | 50 |
| No. of Edges | 695 | 355 | 91 | 140 |
| Positive edges | 590 | 331 | 59 | 92 |
| Negative edges | 105 | 24 | 32 | 48 |
| Average degree | 10.37 | 8.77 | 3.79 | 5.6 |
| Average weighted degree | 9.53 | 9.79 | 4.53 | 2.66 |
| Network diameter | 7 | 7 | 5 | 9 |
| Modularity | 0.64 | 0.51 | 0.63 | 1.37 |
| Connected components | 30 | 34 | 21 | 23 |
| Average clustering coefficient | 0.61 | 0.61 | 0.58 | 0.59 |
| Average path length | 3.11 | 2.80 | 2.27 | 2.91 |

**Table S3** Two-way ANOVA for topological properties of the bacterial and fungal subnetworks

|  |  |  | F-value | P-value |
| --- | --- | --- | --- | --- |
| Bacterial networks | P/N of the whole network | aggregate | 0.357 | 0.572 |
|  |  | treatment | 3.886 | **0.045** |
|  |  | aggregate× treatment | 0.082 | 0.784 |
|  | P/N associated with keystone taxa | aggregate | 4.824 | **0.033** |
|  |  | treatment | 0.385 | 0.558 |
|  |  | aggregate× treatment | 0.305 | 0.601 |
|  | ACC | aggregate | 12.134 | **0.013** |
|  |  | treatment | 17.303 | **0.006** |
|  |  | aggregate× treatment | 0.454 | 0.543 |
| Fungal networks | P/N of the whole network | aggregate | 0.423 | 0.539 |
|  |  | treatment | 12.387 | **0.012** |
|  |  | aggregate× treatment | 0.600 | 0.468 |
|  | P/N associated with keystone taxa | aggregate | 0.836 | 0.395 |
|  |  | treatment | 0.501 | 0.505 |
|  |  | aggregate× treatment | 1.466 | 0.271 |
|  | ACC | aggregate | 1.104 | 0.334 |
|  |  | treatment | 2.980 | 0.135 |
|  |  | aggregate× treatment | 15.665 | **0.007** |

Notes: P/N, positive to negative edges ratio; ACC, average clustering coefficients


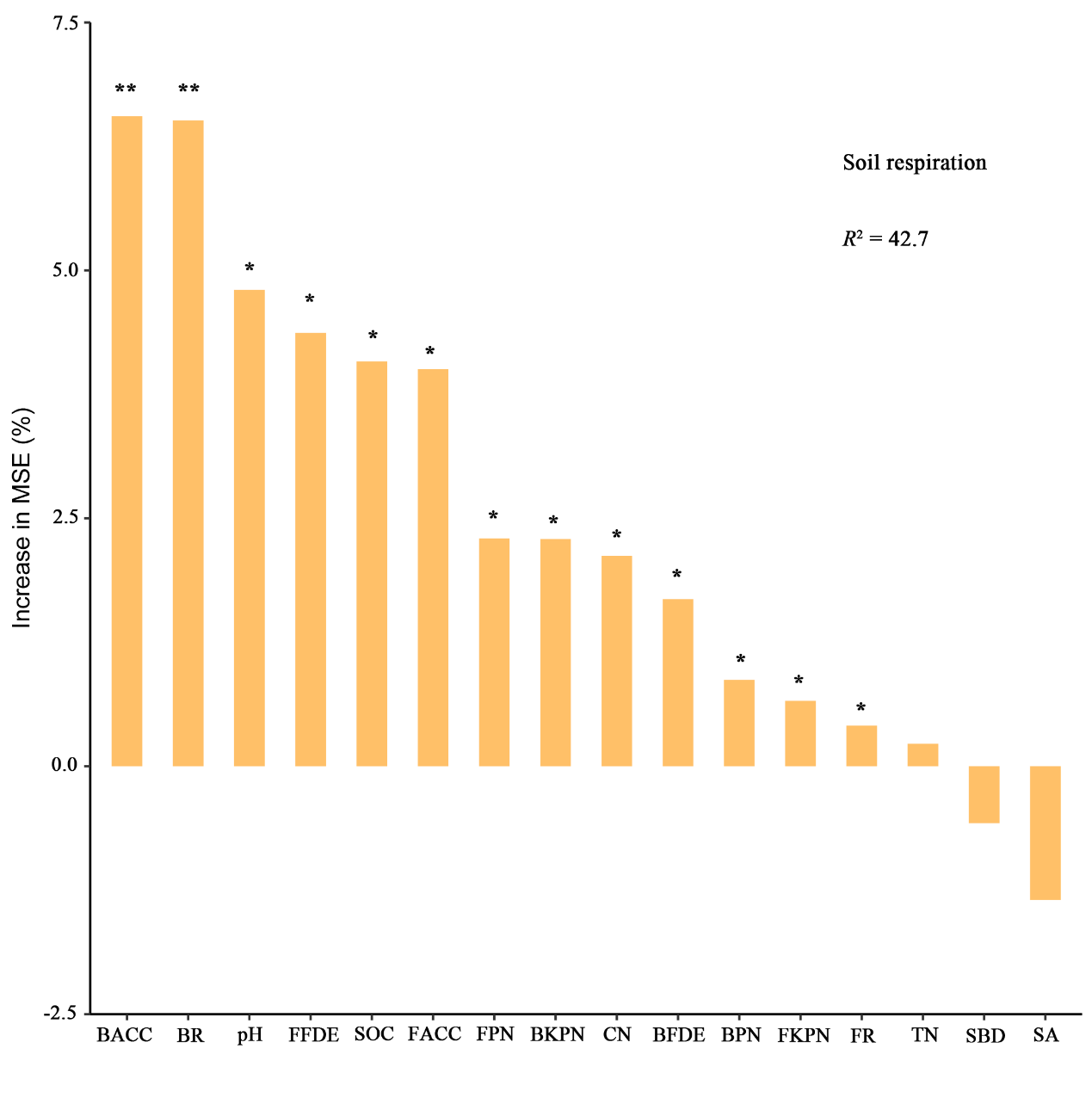


**Figure S1** The importance of soil properties, microbial abundance and community network on soil respiration. Note: MSE, mean square error; SOC, soil organic carbon; TN, soil total nitrogen; SBD, soil bulk density; SA, soil aggregates; C:N, the ratios of total carbon and nitrogen; BPN, P/N of the whole network; BKPN, P/N of bacterial network associated with keystone taxa; BACC, average clustering coefficients of the whole network; BR, relative abundance of bacteria; BFDE, first dominant eigengenes of bacterial community composition; FPN, P/N of the whole network; FKPN, P/N of bacterial network associated with keystone taxa; FACC, average clustering coefficients of fungal network; FR, relative abundance of fungi; FFDE, first dominant eigengenes of fungal community composition.
